# Novel *PAX9* and *COL1A2* Missense Mutations Causing Tooth Agenesis and OI/DGI without Skeletal Abnormalities

**DOI:** 10.1371/journal.pone.0051533

**Published:** 2012-12-05

**Authors:** Shih-Kai Wang, Hui-Chen Chan, Igor Makovey, James P. Simmer, Jan C-C. Hu

**Affiliations:** Department of Biologic and Materials Sciences, University of Michigan School of Dentistry, Ann Arbor, Michigan, United States of America; Sanjay Gandhi Medical Institute, India

## Abstract

Inherited dentin defects are classified into three types of dentinogenesis imperfecta (DGI) and two types of dentin dysplasia (DD). The genetic etiology of DD-I is unknown. Defects in dentin sialophosphoprotein (*DSPP*) cause DD type II and DGI types II and III. DGI type I is the oral manifestation of osteogenesis imperfecta (OI), a systemic disease typically caused by defects in *COL1A1* or *COL1A2*. Mutations in *MSX1*, *PAX9*, *AXIN2*, *EDA* and *WNT10A* can cause non-syndromic familial tooth agenesis. In this study a simplex pattern of clinical dentinogenesis imperfecta juxtaposed with a dominant pattern of hypodontia (mild tooth agenesis) was evaluated, and available family members were recruited. Mutational analyses of the candidate genes for DGI and hypodontia were performed and the results validated. A spontaneous novel mutation in *COL1A2* (c.1171G>A; p.Gly391Ser) causing only dentin defects and a novel mutation in *PAX9* (c.43T>A; p.Phe15Ile) causing hypodontia were identified and correlated with the phenotypic presentations in the family. Bone radiographs of the proband’s dominant leg and foot were within normal limits. We conclude that when no *DSPP* mutation is identified in clinically determined isolated DGI cases, *COL1A1* and *COL1A2* should be considered as candidate genes. *PAX9* mutation p.Phe15Ile within the N-terminal β-hairpin structure of the *PAX9* paired domain causes tooth agenesis.

## Introduction

Osteogenesis imperfecta (OI) is a hereditary, bone fragility disease that varies in severity from mild bone defects to neonatal lethality. Mutations in the type-I collagen genes, *COL1A1* and *COL1A2*, have been identified in approximately 90% of individuals with OI [1]. Dentinogenesis imperfecta type I (DGI-I) is a common phenotypic feature of OI [2]. Non-syndromic dentin defects categorized as dentin dysplasia type II (DD-II), dentinogenesis imperfecta type II (DGI-II) and type III (DGI-III) are generally caused by dominant mutations in dentin sialophosphoprotein (*DSPP*), which encodes the most abundant non-collagenous matrix component of dentin [3]. DSPP is critical for predentin formation and dentin mineralization [4], although the dentin malformations associated with *DSPP* mutations are likely due to odontoblast pathology caused by dominant negative or gain of function effects [5], [6].

Familial tooth agenesis is distinguished from conditions with multiple missing teeth occurring in syndromes, such as ectodermal dysplasia. *MSX1*, *PAX9*, *AXIN2*, *WNT10A*, and *EDA* are proven candidate genes for familial tooth agenesis [7], [8]. In this study we identified a Caucasian family with familial tooth agenesis going back at least three generations, but isolated dentin defects occurred only in the proband. Using a target gene approach, we identified a novel missense mutation in *PAX9* (p.Phe15Ile) that is responsible for the tooth agenesis. Characterization of the *DSPP* 5-prime regulatory region, intron/exon borders, and coding region identified no potential disease-causing mutations. Despite the absence of skeletal abnormalities, mutational analyses of the *COL1A1* and *COL1A2* genes identified a novel *COL1A2* mutation (p.Gly391Ser) only in the proband that explains his dentin phenotype.

## Materials and Methods

### Human subjects

The human study protocol and subject consents were reviewed and approved by the Institutional Review Board at the University of Michigan. Study participants signed appropriate written consents after an explanation of their contents and after their questions about the study were answered. Any minors age 8 or older signed a written assent form after their parent completed a written parental consent for participation of the minor.

**Figure 1:**
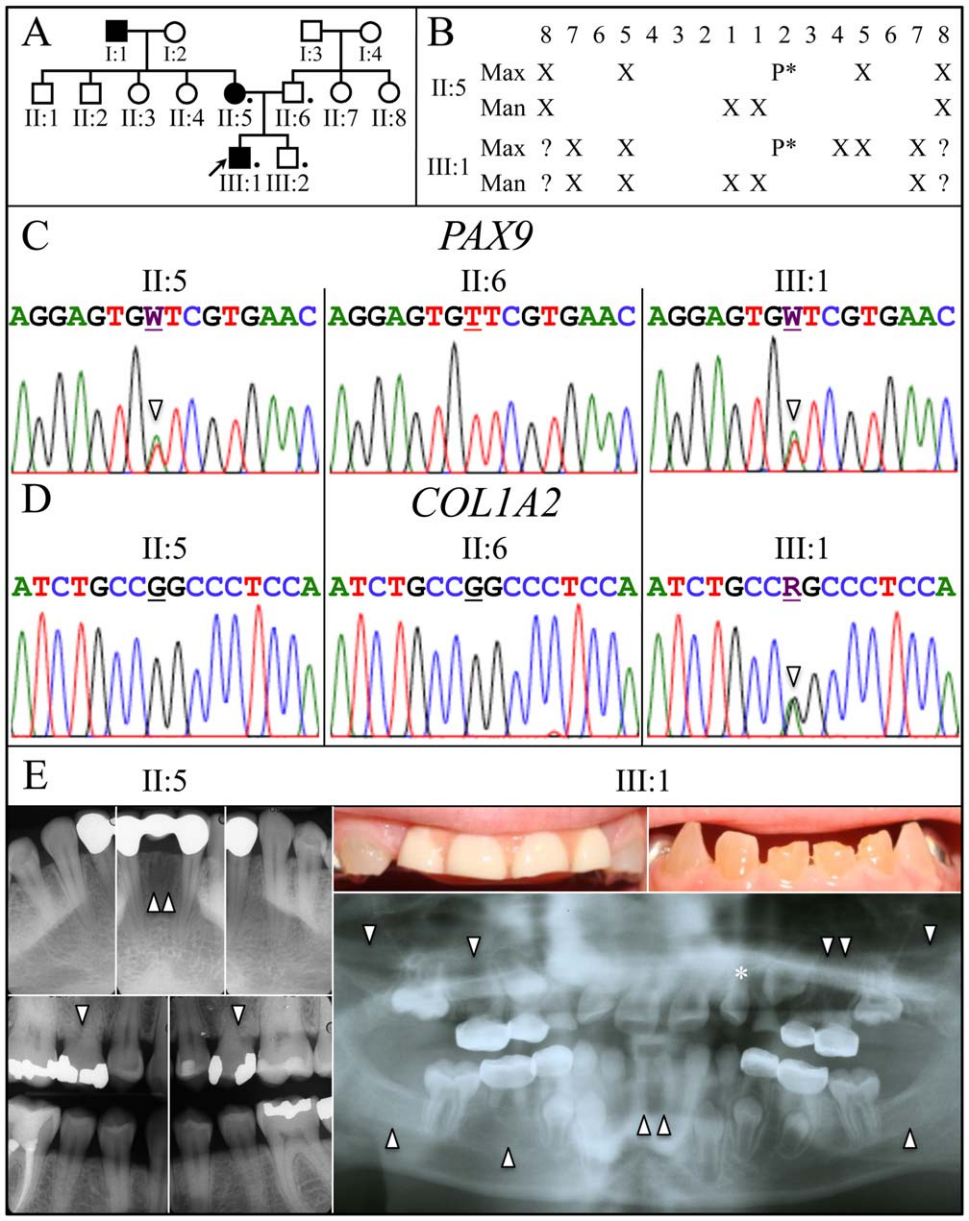
Family pedigree, missing teeth and disease causing mutations. *A:* The family pedigree follows the tooth agenesis trait for 3 generations and is consistent with an autosomal-dominant pattern of inheritance. **Key**: A filled icon indicates tooth agenesis. A dot indicates individual who donated samples. ***B:*** Chart of missing teeth in mother (II:5) and the proband (III:1). ***C:*** DNA sequencing chromatograms show that the affected mother (II:5) and proband (III:1) had a T or A (W) (arrowhead) at position g.5368 (NCBI Ref. Seq. NC_000014.8). This *PAX9* mutation (g.5368T>A; c.43T>A; p.Phe15Ile) caused the tooth agenesis. ***D:*** DNA sequencing chromatograms show both parents (II:5; II:6) had the wild-type G, while the proband had a G or A (R) (arrowhead) at position g.15941 (NCBI Ref. Seq. NG_007405.1). This spontaneous *COL1A2* mutation (g.15941G>A; c.1171G>A; p.Gly391Ser) caused the dentin defects in the proband. ***E:*** Radiographs of the mother (II:5) and proband (III:1) document the missing teeth (arrowheads) and the peg lateral (*) in the proband. Oral photos show the proband’s primary anterior teeth show the brownish discoloration and attrition. His maxillary incisors were removed because of severe attrition and a pediatric partial denture was placed. The proband’s radiographs show the bulbous crowns with cervical constrictions and thin, narrow roots.

A five-year-old boy of European descent was referred by a genetics clinic for evaluation of dentin defects. The proband’s pediatrician and geneticist considered OI, but decided that DGI-II was a better diagnosis due to the lack of systemic signs and history of bone fractures. The proband’s growth and developmental parameters were within normal limits. He was the only family member with dentin defects in the known history of the extended family. Oral photographs and radiographs were obtained from the family’s dental care provider. The dental radiographs revealed the tooth agenesis in the proband and his mother. The mother reported that this trait came from her father and that her siblings were unaffected. Blood samples (5 cc) were collected from the proband, his brother, and their biological parents. Radiographic evaluation of weight-bearing bones on the proband’s dominant side was conducted and a report compiled by the Radiology Department, Spectrum Health, Grand Rapids, Michigan.

**Figure 2:**
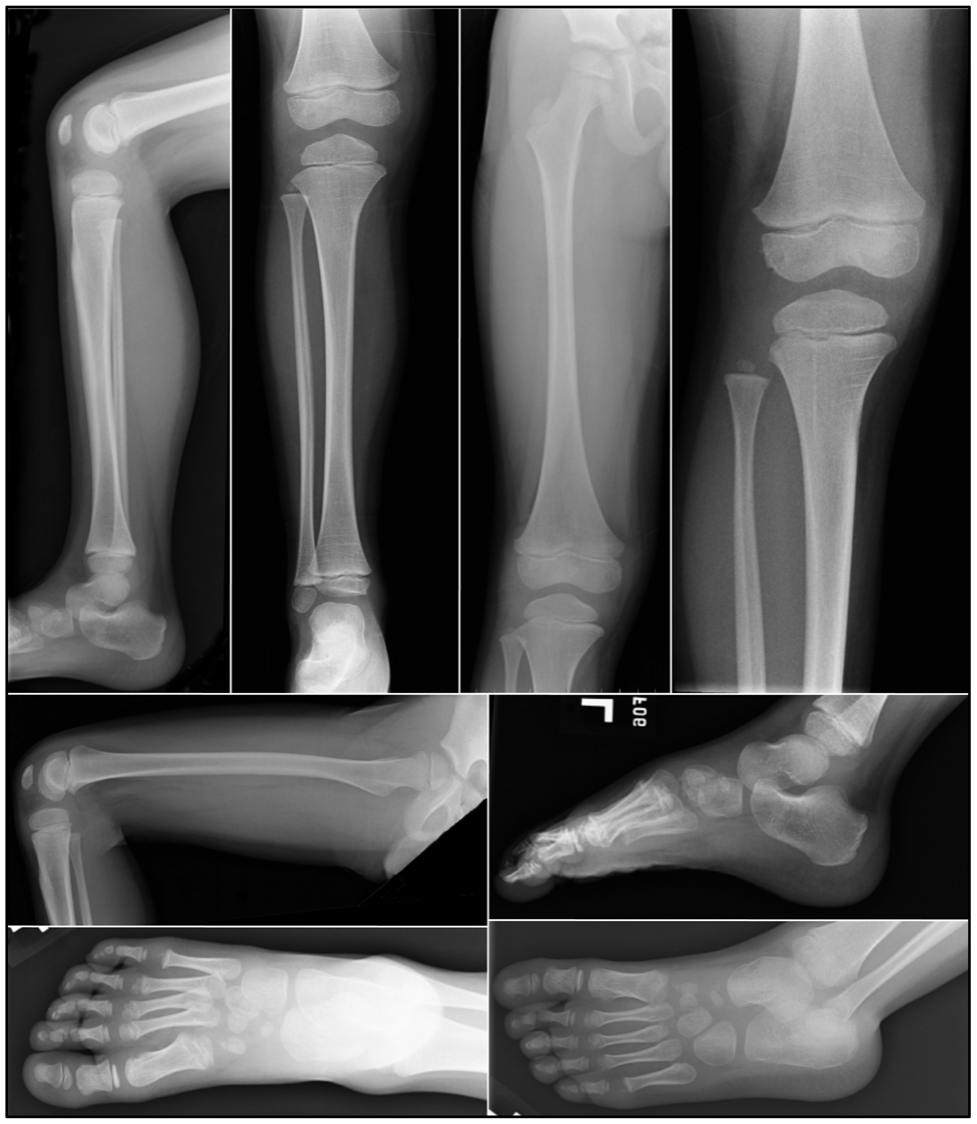
Radiographs of the proband’s left (**dominant**) **leg at age 6.** Radiographs show the femur, tibia, fibula, knee, and foot. No significant osteopenia, bony destructive process, periosteal reactions, or evidence of any acute fractures, dislocations, injuries, or remote traumatic changes were observed.

### Mutational analysis

The *MSX1* and *PAX9* coding exons and intron junctions were amplified by PCR as previously reported [9]. The amplification products were purified and characterized by direct DNA sequencing at the University of Michigan DNA Sequencing Core. Because the dentin defects were observed only in the proband, fifteen polymorphic markers were amplified using maternal, paternal and proband DNA for paternity testing. In assessing the *DSPP* sequence, the putative promoter region (1–2000 bp upstream to the transcription start site) and the first four exons were analyzed by PCR amplification and direct DNA sequencing. *DSPP* exon 5 was characterized by cloning and sequencing due to the highly variable repeat region of *DPP*. The PCR primers for *DPP* (DSPP-FRF 5′-AGTCCATGCAAGGAGATGATCC-3′ and DSPP-FRR 5′-CTAATCATCACTGGTTGAGTGG-3′) annealed at 57°C and generated a 2534-bp amplification product that was subcloned (TOPO cloning kit, Invitrogen, Calsbad, CA). Twenty clones from two separate PCR amplifications were sequenced using forward and reverse M13 primers and two custom primers (5′-CAGACAGCAGCAAATCAGAG-3′, 5′-GATAGCGACAGCAGCAATAGA-3′). The *COL1A1* and *COL1A2* coding sequences were characterized by Athena Diagnostics (Worcester, MA, USA). The putative *PAX9* and *COL1A2* disease causing mutations were analyzed using PolyPhen-2 (Polymorphism Phenotyping version 2.2.2) [10].

**Table 1:** *PAX9* mutations causing familial tooth agenesis.

	cDNA	Protein	Reference
1)	*PAX9* deletion	p.0	[21]
2)	c.1A>G;	p.M1V	[22]
3)	c.16G>A	p.G6R	[19]
4)	c.43T>A	p.F15I	This Report
5)	c.62T>C	p.L21P	[23]
6)	c.76C>T	p.R26W	[24]
7)	c.83>C	p.R28P	[25]
8)	c.109_110insG	p.I37Sfs*41	[26]
9)	c.128G>A	p.S43K	[19]
10)	c.139C>T	p.R47W	[27]
11)	c.151G>C	p.G51S	[28]
12)	c.175C>T	p.R59*	[29]
13)	c.175_176ins288	p.R59Zfs*177	[23]
14)	c.218_219insG	p.S74Qfs*317	[30]
15)	c.259A>T	p.I87F	[31]
16)	c.271A>G	p.K91E	[23]
17)	c.340A>T	p.K114*	[32]
18)	c.433C>T	p.Q145*	[33]
19)	c.503C>G	p.A168G	[34]
20)	c.619_621delATCins24bp	p.I207Yfs*211	[28]
21)	c.793insC	p.V265RfsX315	[35]

## Results

### Pedigree analyses

A 3-generation pedigree was constructed (Fig 1A) based upon family histories provided by the mother and maternal grandmother, and was consistent with an autosomal dominant pattern of inheritance for tooth agenesis. Dentin defects occurred only in the proband.

**Table 2:** Collagen (*COL1A1* and *COL1A2*) sequence variations identified in the proband.

*COL1A1*				
cDNA	Protein	Zygosity	OI Phenotype	Reference
c.299–20C>G	NA	homozygous	Not associated	This report
c.3223A>G	p.T1075A	homozygous	Not associated	[36]
c.2560–18C>G	NA	heterozygous	Not associated	[37]
C.4249–12G>A	NA	heterozygous	Not associated	This report

*COL1A2*				
cDNA	Protein	Zygosity	OI Phenotype	Reference
c.1171G>A	p.G391S	heterozygous	Dominant DGI	This report
c.594+118delT	NA	heterozygous	Not associated	This report
c.1645C>G	p.P549A	homozygous	Not associated	[38]
c.936+14C>T	NA	homozygous	Not associated	This report

### Clinical and radiographic findings

The maternal grandfather, mother and the proband exhibited tooth agenesis. The mother was missing tooth numbers 4, 13, 24, 25, and all third molars (Fig 1B, 1E). In both the mother and the proband, tooth 10 was a peg lateral incisor. The proband at age 8.0 showed no radiographic evidence of developing tooth germs for tooth numbers 2, 5, 12, 13, 15, 18, 24, 25, 29, 31 and was too young to determine the status of his third molars (Fig 1B, 1E). His primary teeth had bulbous crowns, enlarged pulp chambers, and opalescent dentin consistent with the phenotypic features of dentinogenesis imperfecta, while none of those features were observed in the radiographs of his brother, mother and father (data not shown). Radiographs of the dominant (left) leg of the proband (Fig 2), including his femur, tibia, fibula, foot, and knee, revealed no significant osteopenia, bony destructive process, periosteal reactions, or evidence of any acute fractures, dislocations, injuries, or remote traumatic changes. Thus there was no evidence supporting a diagnosis of osteogenesis imperfecta.

### Identification of a *PAX9* and a *COL1A2* missense mutation

The proband and his mother had a novel missense mutation (c.43T>A; p.Phe15Ile) in *PAX9* (Fig 1C) that was not in unaffected family members, the dbSNP database or in 1000 Genomes Project Pilot Data [11]. No potential disease-causing mutations were identified in *MSX1.* The mutated amino acid is invariant in vertebrates and the p.Phe15Ile substitution was predicted to be probably damaging by PolyPhen-2 analyses. Supported by the finding of other *PAX9* mutations causing familial tooth agenesis (Table 1), we conclude that this mutation causes the tooth agenesis in this family.

No potential disease-causing mutations were identified in the promoter, coding regions, or intron/exon boundaries in *DSPP*. Analyses covering all intron/exon junctions of *COL1A1* identified only known polymorphisms (Table 2). A novel missense mutation (c.1171G>A; p.Gly391Ser) in one *COL1A2* allele only in the proband was identified. This sequence variation is not reported in the SNP database. As paternity was confirmed, this was likely a spontaneous germ-line mutation. The mutated amino acid is invariant in vertebrates and the p.Gly391Ser substitution was predicted to be probably damaging by PolyPhen-2 analyses. Supported by Pallos *et* *al*, documenting a family with dentinogenesis imperfecta associated with a minimal bone phenotype caused by a *COL1A1* mutation [12], we conclude that the spontaneous *COL1A2* mutation caused the proband’s dentin defects.

## Discussion

The objective of this study was to identify the causative gene mutation (s) for the dentin defects and familial tooth agenesis in this family. The dentin defects were caused by a novel spontaneous missense mutation that substituted serine for a conserved glycine in the triple helical region of COL1A2. The familial tooth agenesis segregated as an autosomal dominant trait and resulted from a novel missense mutation affecting the conserved N-terminal DNA binding domain of PAX9.

### *COL1A2* p.G391S mutation and OI/DGI

Type-I collagen, the major extracellular matrix protein of bone, dentin, and skin, is comprised of two pro-á1 chains and one pro-á2 chain encoded by *COL1A1* and *COL1A2,* respectively. Mutations in these two genes generally cause dominant forms of OI, characterized by bone fragility, altered sclera hue, hearing loss, dentin defects, and soft tissue dysplasia. However, the clinical presentation of OI is highly heterogeneous. Since 85% of organic component in tooth dentin is type-I collagen, dentin defects are observed in many OI cases. Pallos *et* *al* reported a family with autosomal-dominant OI in which affected members exhibited dentin defects without obvious bone abnormalities, except hyperextensible joints and joint pain. After checking *DSPP*, they identified the disease-causing mutation in *COL1A1* [12]. Our investigation followed a similar course. After finding no potential disease-causing mutations in *DSPP*, we analyzed *COL1A1* and *COL1A2* and identified the missense mutation (p.Gly391Ser) in *COL1A2*. We then searched more carefully for bone defects. Radiographs of the weight-bearing bones on the proband’s dominant side revealed no pathologic bone defects. Based upon Pallos’ report and our present study, we propose that *COL1A1* and *COL1A2* should be regarded as strong candidate genes for isolated dentin defects when no mutation can be identified in *DSPP*.

The repeating Gly-X-Y sequence motif in collagen is critical for its triple helical conformation. Glycine must occupy every third position as its side chain is the only one small enough to fit into the interior position of the helix. Substitutions for invariant glycines are the most common cause of clinically significant OI [13]. The clinical severity of different glycine mutations varies significantly from lethality to a mild connective tissue disorder, depending upon the location in the chain where the glycine substitution occurs. Several models have been developed to correlate glycine mutations with the severity of OI phenotypes. Bodian *et* *al* proposed a model to predict the clinical lethality of collagen glycine mutations [14] and Rauch *et* *al* provided a detailed picture of genotype–phenotype correlations in OI patients with glycine mutations [2]. Besides affecting the helix, crucial regions may represent certain specific ligand-binding sites [15]. Finding that the p.Gly391Ser substitution in the pro-á2 chain, leads to dentin defects without a clinically-detectable skeletal phenotype suggests that this glycine may reside in a region that interacts with DSPP.

### *PAX9* p.F15I mutation and familial tooth agenesis

Tooth formation is a sequential process of epithelial-mesenchymal interactions controlled by numerous molecules and signaling pathways [16]. Mutations in genes critical for the early stages of tooth formation lead to familial tooth agenesis. At present, mutations causing non-syndromic tooth agenesis have been identified in *MSX1*, *PAX9*, *AXIN2*, *EDA,* and *WNT10A* [7]. In general, the pattern of missing teeth correlates with the causative gene. Second bicuspids and third molars are frequently involved in *MSX1*-associated tooth agenesis. *PAX9* mutations lead to tooth agenesis of second bicuspids, second molars, and some central incisors [9]. *AXIN2* aberrations cause multiple missing teeth, intestinal polyposis, and predispose to colorectal cancer [17]. *EDA*-associated tooth agenesis is more likely to miss multiple anterior teeth [18]. In the present study, agenesis of third molars, second bicuspids, and mandibular central incisors in the mother, suggested *MSX1*-associated tooth agenesis. No *MSX1* mutation could be identified, but a *PAX9* missense mutation substituting isoleucine for a highly conserved phenylalanine (p.Phe15Ile) was identified in the paired box domain crucial for DNA binding. Wang *et* *al* identified a *PAX9* mutation (p.Gly6Arg) causing a mild hypodontia phenotype almost identical to that of our proband’s mother [19]. Interestingly, our p.Phe15Ile substitution is structurally close to the p.Gly6Arg mutation. Both of these amino acids reside in the N-terminal beta hairpin of the paired domain [20]. Initially this suggested to us that substitutions within the N-terminal beta hairpin of PAX9 cause a relatively minor functional deficit. However, our proband had substantially more missing teeth than his mother, highlighting the variable expressivity of the tooth agenesis phenotype. Characterizing additional *PAX9* mutations affecting the N-terminal beta hairpin of the paired domain and documenting their associated dental phenotypes will be necessary before we can conclude that such substitutions generally cause a minor functional deficit.
